# Long COVID-19: Rethinking mental health

**DOI:** 10.1016/j.clinsp.2022.100067

**Published:** 2022-06-13

**Authors:** Miguel Gallegos, Nelson Portillo, Pablo Martino, Mauricio Cervigni

**Affiliations:** a Facultad de Ciencias de la Salud. Universidad Católica del Maule, Talca, Chile; b Facultad de Psicología. Universidad Nacional de Rosario, Argentina; c Programa de Pós-Graduação em Psicología, Pontificia Universidade Católica de Minas Gerais, Belo Horizonte, MG, Brazil; d Consejo Nacional de Investigaciones Científicas y Técnicas, Buenos Aires, Argentina; e Centro de Investigación en Neurociencias de Rosario, Facultad de Psicología. Universidad Nacional de Rosario, Argentina

As the pandemic enters its third year, the long-term effects of the SARS-CoV-2 virus infection on people's health are beginning to be better understood. In particular, scientists have identified several symptoms that are likely to persist for a few weeks or even months. These symptoms are not only physiological, pulmonary, cardiovascular, muscular, and the like, but also psychological, cognitive, and neuropsychiatric.^1^, ^2^, ^3^, ^4^ The World Health Organization provided a special classification of the disease in September 2020, and a clinical definition in October 2021.

“Post COVID-19 condition occurs in individuals with a history of probable or confirmed SARS-CoV-2 infection, usually 3-months from the onset of COVID-19 with symptoms that last for at least 2-months and cannot be explained by an alternative diagnosis. Common symptoms include fatigue, shortness of breath, cognitive dysfunction but also others and generally have an impact on everyday functioning. Symptoms may be new-onset following initial recovery from an acute COVID-19 episode or persist from the initial illness. Symptoms may also fluctuate or relapse over time. A separate definition may be applicable for children”.^5^

Although this is a provisional definition derived from a consultation with international experts, it does not sufficiently address a number of elements. For example, it is not specific with respect to children, nor does it adequately cover other special populations, such as racial and ethnic minorities, persons with disabilities, institutionalized persons, etc. Nor does it adequately address mental health matters, which have been severely affected by the conditions imposed during the pandemic. Table 1 summarizes specific information related to mental health extracted from three studies,^2^, ^3^, ^4^ which described the general symptoms of long COVID-19.

**Table 1:** Prevalence of psychological, cognitive, and neuropsychiatric symptoms by long COVID-19.

Clinical manifestations^2^	Clinical manifestations^3^	Clinical manifestations^4^
Health care related mental health Psychiatric illness Attention disorder Memory loss Anxiety Depression Sleep disorder Sleep apnea Dizziness Mood disorder Dysphoria Paranoia Obsessive compulsive disorder Post-traumatic stress disorder	Psychological: Post-traumatic stress disorder Distress Anxiety Depression Anxiety or depression Dementia/memory loss Obsessive compulsive disorder Panic attacks Psychiatric morbidity Emotional symptoms Low QoL Dysphoria Anorexia *Cognitive:* Loss of attention Confusion Neurocognitive impairment Concentration deficits Short-term memory deficits Word finding difficulty	Neuropsychiatric: Acute (sudden) confusion/disorientation Changes to sense of smell and taste Dizziness, unsteadiness, or balance issues Hallucinations Headaches and related symptoms Insomnia Other sleeping symptoms Sleep apnea Slurring words/speech All sensorimotor symptoms Brain fog Memory issue Neuralgia (never pain) Speech/Language issues Tremors

Although studies differ in the symptoms reported, a set of characteristic symptoms can serve to guide clinical care not only in post-hospitalized patients but also in outpatients. It is important for professionals to keep in mind that the long-term effects of COVID-19 on mental health vary and the prevalence of symptoms is also very heterogeneous. In addition, the effects may involve different organs and systems, causing repercussions on the ability to work, perform daily activities, and quality of life, in general.

For many, COVID-19 will not end after being discharged from a hospital or by testing negative on a PCR test. Instead, the fallout will continue, and a number of symptoms are likely to be experienced for some time. Some studies have already offered recommendations for the primary care of certain persistent neuropsychiatric symptoms,^6^ but greater precision is still required on all the possible effects as well as for their appropriate therapeutic approach.

In a study that evaluated post-hospitalized patients between two- and seven months post-discharge, researchers found that among the 10 most frequent symptoms were the following: muscle pain, fatigue, physical slowing, poor sleep quality, joint pain or swelling, limb weakness, shortness of breath, pain, short-term memory loss and slowed thinking. The study also reported that 25% of the cohort manifested persistent symptoms of anxiety and depression, and 12.2% manifested post-traumatic stress disorder.^7^

The long-term effects of COVID-19 on people's cognitive performance are a source of concern because of the implications for people's daily activities. In a study focused on long-term cognitive alterations, 77.8% had difficulty concentrating, 69% reported brain fog, 67.5% reported forgetfulness, 59.5% experienced word-finding problems (ToT), and 43.7% reported semantic dysfluency (saying or writing words incorrectly). In addition, 54.6% failed to return to work due to ongoing symptoms, 34.5% lost their jobs, and 17.6% reported financial difficulties. Other findings from the same study showed that close to two-thirds experienced difficulties in carrying out daily activities and half said that physicians did not take their health problems seriously.^8^

The information gathered so far should serve as a starting point for rethinking the long-term effects of COVID-19, but the authors also need a deeper understanding of the effects of the pandemic on people's mental health.^9^ While many studies have been concerned with documenting the most persistent symptoms on people's mental health, few have questioned the extent to which these symptoms are strictly the consequence of COVID-19 infections. A causal relationship is taken for granted but is not sufficiently established, even though certain physiological and neurobiological implications of COVID-19 disease on people's health are recognized.

When establishing any causal effects of COVID-19 infection and mental health outcomes, the authors also need to recognize the role played by social and structural determinants of health. Since the COVID-19 pandemic has disproportionally affected communities of color and low-income populations, assessments of mental health outcomes need to account for any disparities, inequities, and gaps that may partially explain or exacerbate them. A recent rapid review that synthesized 42 studies has shown that certain social determinants of health matter when studying COVID-19 outcomes, although the evidence is still limited for a number of social determinants.^10^

The COVID-19 pandemic and its effects should put the focus back on the importance of mental health professionals in establishing the differential diagnosis, monitoring the mental health of patients, and the most appropriate therapeutic course of action. It is also important to underscore the concept that health is much more than the mere absence of disease, and therefore, it is essential to have professionals who know how to adequately address mental health problems and their implications for people's lives.

## Authors' contributions

Miguel Gallegos: Contributed to the design, data analysis, interpretation, and writing of first and subsequent drafts of the paper.

Nelson Portillo: Contributed to the data analysis, interpretation, and writing of first and subsequent drafts of the paper.

Pablo Martino: Contributed to the data analysis, interpretation, and writing of first and subsequent drafts of the paper.

Mauricio Cervigni: Contributed to the interpretation and writing of first and subsequent drafts of the paper.

## Conflicts of interest

The authors declare no conflicts of interest.
